# Supplementary material for: Release of Interferon-β (IFN-β) from Probiotic Limosilactobacillus reuteri-IFN-β (LR-IFN-β) Mitigates Gastrointestinal Acute Radiation Syndrome (GI-ARS) following Whole Abdominal Irradiation
Source: Cancers (Basel). 2023 Mar 8;15(6):1670. doi: 10.3390/cancers15061670 (PMC10046795; doi:10.3390/cancers15061670)

Supplemental Figure S1. Luminex Assay demonstrating significant changes in protein expression of LR-IFN-β in the intestine following 12 Gy TBI.

group	Intestine G CSF
ANOVA F-test p-value	0.0084
Control	12.7±6.2 (n=4)
IR	160.1±73.2 (n=4)<br>**p=0.0130 vs control**
IR + IFN-B	76.0±12.9 (n=4)<br>**p=0.5028 vs control**<br>**p=0.2458 vs IR**
IR + LR	157.9±68.5 (n=4)<br>**p=0.0145 vs control**<br>**p=1.0000 vs IR**<br>**p=0.2668 vs IR + IFN-B**
IR + LR IFN-B	120.6±69.7 (n=4)<br>**p=0.0885 vs control**<br>**p=0.8441 vs IR**<br>**p=0.7801 vs IR + IFN-B**<br>**p=0.8680 vs IR + LR**

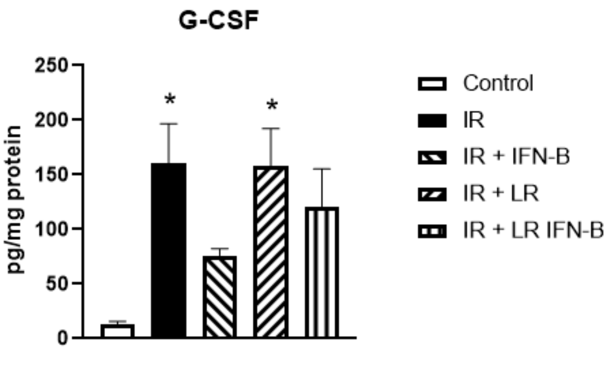

*p < 0.0150 compared to control

Supplemental Figure S2. Luminex assay demonstrating significant changes in protein expression of LIF in the intestine following 12 Gy TBI.

group	Intestine LIF
ANOVA F-test p-value	<0.0001
Control	23.0±10.0 (n=4)
IR	129.9±26.1 (n=4)<br>**p<0.0001 vs control**
IR + IFN-B	80.3±25.0 (n=4)<br>**p=0.0073 vs control**<br>**p=0.0198 vs IR**
IR + LR	73.8±11.1 (n=3)<br>**p=0.0283 vs control**<br>**p=0.0145 vs IR**<br>**p=0.9910 vs IR + IFN-B**
IR + LR IFN-B	106.2±12.1 (n=3)<br>**p=0.0006 vs control**<br>**p=0.5083 vs IR**<br>**p=0.4285 vs IR + IFN-B**<br>**p=0.2877 vs IR + LR**

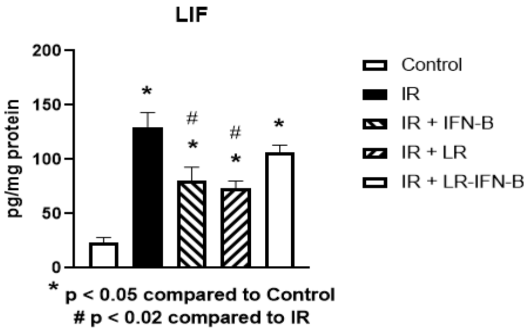

Supplemental Figure S3. Luminex assay demonstrating significant changes in protein expression of M-CSF in the intestine following 12 Gy TBI.

group	Intestine M CSF
ANOVA F-test p-value	0.0115
Control	10.3±5.7 (n=4)
IR	22.9±4.5 (n=4)<br>**p=0.0105 vs control**
IR + IFN-B	12.8±3.8 (n=4)<br>**p=0.9312 vs control**<br>**p=0.0450 vs IR**
IR + LR	12.6±4.0 (n=4)<br>**p=0.9421 vs control**<br>**p=0.0419 vs IR**<br>**p=1.0000 vs IR + IFN-B**
IR + LR IFN-B	17.2±4.3 (n=3)<br>**p=0.3120 vs LGR contro**<br>**p=0.4888 vs IR**<br>**p=0.7060 vs IR + IFN-B**<br>**p=0.6853 vs IR + LR**

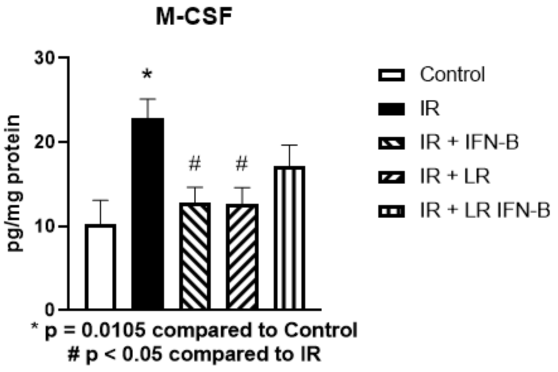

Supplemental Figure S4. Luminex assay demonstrating significant changes in protein expression of MIG in the intestine following 12 Gy TBI.

group	Intestine MIG
ANOVA F-test p-value	0.0007
Control	1169.0±599.2 (n=3)
IR	8489.4±924.9 (n=3)<br>**p=0.0020 vs control**
IR + IFN-B	7900.6±1291.6 (n=4)<br>**p=0.0023 vs control**<br>**p=0.9922 vs IR**
IR + LR	3873.9±1227.5 (n=4)<br>**p=0.3363 vs control**<br>**p=0.0354 vs IR**<br>**p=0.0498 vs IR + IFN-B**
IR + LR IFN-B	7510.5±3188.8 (n=4)<br>**p=0.0038 vs control**<br>**p=0.9507 vs IR**<br>**p=0.9979 vs IR + IFN-B**<br>**p=0.0846 vs IR + LR**

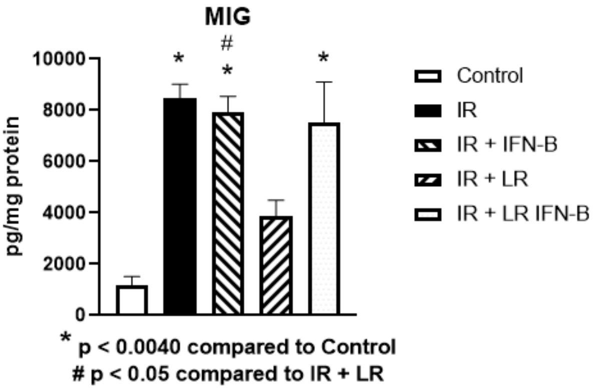

Supplemental Figure S5: Luminex assay demonstrating significant changes in protein expression of TNF-α in the intestine following 12 Gy TBI.

group	Intestine TNF a
ANOVA F-test p-value	0.0081
Control	14.2±6.3 (n=4)
IR	30.3±6.6 (n=4)<br>**p=0.0108 vs control**
IR + IFN-B	20.3±2.4 (n=4)<br>**p=0.5997 vs control**<br>**p=0.1611 vs IR**
IR + LR	28.9±5.9 (n=4)<br>**p=0.0210 vs control**<br>**p=0.9965 vs IR**<br>**p=0.2776 vs IR + IFN-B**
IR + LR IFN-B	20.9±6.9 (n=4)<br>**p=0.5028 vs control**<br>**p=0.2106 vs IR**<br>**p=0.9998 vs IR + IFN-B**<br>**p=0.3512 vs IR + LR**

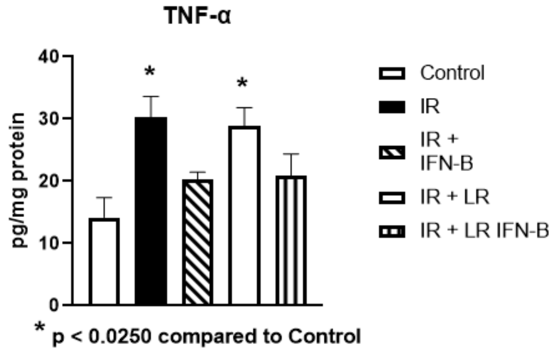

Supplemental Figure S6. Luminex assay demonstrating significant changes in protein expression of G-CSF in the plasma following 12 Gy TBI.

group	Plasma G CSF
ANOVA F-test p-value	<0.0001
Control	379.7±45.0 (n=3)
IR	12973.5±991.4 (n=3)<br>**p<0.0001 vs control**
IR + IFN-B	10019.2±1496.8 (n=4)<br>**p<0.0001 vs control**<br>**p=0.0567 vs IR**
IR + LR	12021.3±1194.1 (n=4)<br>**p<0.0001 vs control**<br>**p=0.8545 vs IR**<br>**p=0.2213 vs IR + IFN-B**
IR + LR IFN-B	9544.9±1589.8 (n=4)<br>**p<0.0001 vs control**<br>**p=0.0236 vs IR**<br>**p=0.9821 vs IR + IFN-B**<br>**p=0.0937 vs IR + LR**

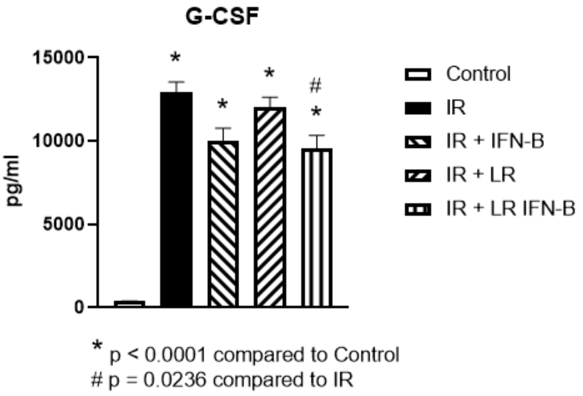

Supplemental Figure S7. Luminex assay demonstrating significant changes in protein expression of Eotaxin in the plasma following 12 Gy TBI.

group	Plasma Eotaxin
ANOVA F-test p-value	<0.0001
Control	1857.3±693.3 (n=4)
IR	13317.3±1944.3 (n=4)<br>**p<0.0001 vs control**
IR + IFN-B	9218.6±705.4 (n=4)<br>**p<0.0001 vs control**<br>**p=0.0009 vs IR**
IR + LR	8651.3±518.0 (n=3)<br>**p<0.0001 vs control**<br>**p=0.0006 vs IR**<br>**p=0.9581 vs IR + IFN-B**
IR + LR IFN-B	11337.3±815.7 (n=4)<br>**p<0.0001 vs control**<br>**p=0.1334 vs IR**<br>**p=0.0984 vs IR + IFN-B**<br>**p=0.0422 vs IR + LR**

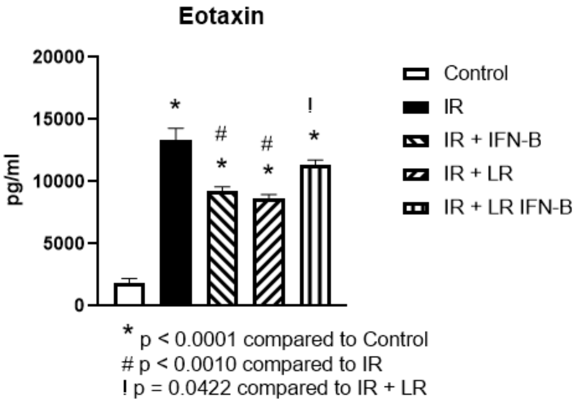

Supplemental Figure S8. Luminex assay demonstrating significant changes in protein expression of Lix in the plasma following 12 Gy TBI.

group	Plasma LIX
ANOVA F-test p-value	0.0023
Control	108.0±55.1 (n=3)
IR	217.0±116.0 (n=2)<br>**p=0.9963 vs control**
IR + IFN-B	1480.4±301.6 (n=3)<br>**p=**0.0044 **vs control**<br>**p=**0.0159 **vs IR**
IR + LR	1024.6±493.2 (n=4)<br>**p=**0.0363 **vs control**<br>**p=0.1206 vs IR**<br>**p=0.4547 vs IR + IFN-B**
IR + LR IFN-B	1262.3±351.0 (n=3)<br>**p=**0.0139 **vs control**<br>**p=**0.0466 **vs IR**<br>**p=0.9312 vs IR + IFN-B**<br>**p=0.8880 vs IR + LR**

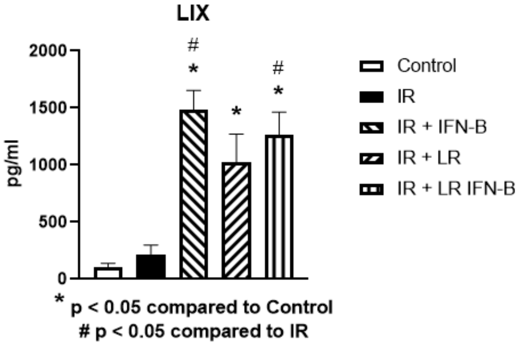

Supplement: Supplementary file 1 [file cancers-15-01670-s001.zip › cancers-2142141-supplementary.pdf]
